# Cardiovascular disease risk prediction by the American College of Cardiology (ACC)/American Heart Association (AHA) Atherosclerotic Cardiovascular Disease (ASCVD) risk score among HIV-infected patients in sub-Saharan Africa

**DOI:** 10.1371/journal.pone.0172897

**Published:** 2017-02-24

**Authors:** Mosepele Mosepele, Linda C. Hemphill, Tommy Palai, Isaac Nkele, Kara Bennett, Shahin Lockman, Virginia A. Triant

**Affiliations:** 1 Department of Medicine, Faculty of Medicine, University of Botswana, Gaborone, Botswana; 2 The Heart Center, Division of Cardiology, Massachusetts General Hospital & Harvard Medical School, Boston, Massachusetts, United States of America; 3 Botswana-Harvard AIDS Institute Partnership, Gaborone, Botswana; 4 Bennett Statistical Consulting Inc, Ballston Lake, New York, United States of America; 5 Division of Infectious Diseases, Brigham & Women`s Hospital, Boston, Massachusetts, United States of America; 6 Department of Immunology & Infectious Diseases, Harvard T. H. Chan School of Public Health, Boston, Massachusetts, United States of America; 7 Division of General Internal Medicine & Division of Infectious Diseases, Massachusetts General Hospital & Harvard Medical School, Boston, Massachusetts, United States of America; Azienda Ospedaliera Universitaria di Perugia, ITALY

## Abstract

**Objectives:**

HIV-infected patients are at increased risk for cardiovascular disease (CVD). However, general population CVD risk prediction equations that identify HIV-infected patients at elevated risk have not been widely assessed in sub-Saharan African (SSA).

**Methods:**

HIV-infected adults from 30–50 years of age with documented viral suppression were enrolled into a cross-sectional study in Gaborone, Botswana. Participants were screened for CVD risk factors. Bilateral carotid intima-media thickness (cIMT) was measured and 10-year predicted risk of cardiovascular disease was calculated using the Pooled Cohorts Equation for atherosclerotic CVD (ASCVD) and the 2008 Framingham Risk Score (FRS) (National Cholesterol Education Program III–NCEP III). ASCVD ≥7.5%, FRS ≥10%, and cIMT≥75^th^ percentile were considered elevated risk for CVD. Agreement in classification of participants as high-risk for CVD by cIMT and FRS or ASCVD risk score was assessed using McNemar`s Test. The optimal cIMT cut off-point that matched ASCVD predicted risk of ≥7.5% was assessed using Youden’s J index.

**Results:**

Among 208 HIV-infected patients (female: 55%, mean age 38 years), 78 (38%) met criteria for ASCVD calculation versus 130 (62%) who did not meet the criteria. ASCVD classified more participants as having elevated CVD risk than FRS (14.1% versus 2.6%, McNemar’s exact test p = 0.01), while also classifying similar proportion of participants as having elevated CVD like cIMT (14.1% versus 19.2%, McNemar’s exact test p = 0.34). Youden’s J calculated the optimal cut point at the 81^st^ percentile for cIMT to correspond to an ASCVD score ≥7.5% (sensitivity = 72.7% and specificity = 88.1% with area under the curve for the receiver operating characteristic [AUC] of 0.82, 95% Mann-Whitney CI: 0.66–0.99).

**Conclusion:**

While the ASCVD risk score classified more patients at elevated CVD risk than FRS, ASCVD score classified similar proportion of patients as high risk when compared with established subclinical atherosclerosis. However, potential CVD risk category misclassification by established equations such as ASCVD may still exist among HIV-infected patients; hence there is still a need for development of a CVD risk prediction equation tailored to HIV-infected patients in SSA.

## Background

Cardiovascular disease (CVD) risk is elevated in patients with HIV [1–3], and emerging data suggest that HIV-infected patients in sub-Saharan Africa (SSA) confront a similarly increased burden of CVD [4–6]. However, identifying individual HIV-infected patients who are at increased risk and candidates for primary CVD risk prevention remains a challenge [7–9]. In most studies, available general population CVD prediction equations are applied among HIV-infected patients to predict risk of hard end-points such as myocardial infarction or stroke. In some instances, the equations are compared to each other in the same clinical cohort to assess agreement in classifying patients as either low or high CVD risk. For instance, studies that applied the 2013 American College of Cardiology (ACC)/American Heart Association (AHA) risk prediction tool for atherosclerotic CVD (ASCVD) among HIV-infected patients have reported mixed results. In some HIV-cohorts, ASCVD underestimated CVD risk as compared to actual occurrence of CVD end-points [10, 11] or presence of high risk morphology plaque [12]. Other studies have demonstrated good [13] versus poor [14] agreement with the well-established Framingham Risk Score (FRS). The FRS has been reported to accurately predict CVD risk [10, 15], underestimate CVD risk [12, 14] or overestimate risk [15]. When applied to HIV cohorts, the HIV-specific CVD risk prediction equation from the Data Collection on Adverse Events of Anti-HIV Drugs (D:A:D) cohort has also been noted to both underestimate predicted CVD risk [10, 11] and overestimate it [15] based on expected CVD endpoints.

Given these mixed data on performance of CVD risk prediction rules across different HIV-infected populations, surrogate CVD end points such as carotid atherosclerosis as assessed by carotid intima-media thickness (cIMT) have been used as an alternate approach to estimate risk of CVD [16, 17] with a cIMT cut-off of ≥75^th^ percentile indicating high CVD risk [18]. Importantly, cIMT has been used as a surrogate CVD endpoint to assess the new ASCVD risk score performance in new populations without their own validated CVD risk prediction scores [19, 20]. The use of cIMT as a surrogate CVD end-point is of particular interest in an African HIV-infected patient population because cIMT is a stronger predictor of stroke than myocardial infarction [21] and HIV-infected patients in SSA experience strokes more frequently than myocardial infarctions [5, 22–24]. We therefore sought to assess correlation between estimated 10 year risk of CVD using the ASCVD risk score or Framingham Risk Score (FRS) with cIMT as a surrogate CVD end-point among virally suppressed HIV-infected patients in Botswana.

## Methods

HIV-infected adults between 30–50 years of age with documented viral suppression were enrolled into a cross-sectional study in Gaborone, Botswana. All participants were screened for the following CVD risk factors (CVDRF): bilateral carotid intima media thickness (cIMT), elevated blood pressure or use of anti-hypertensive treatment, weight, height, waist circumference, cigarette smoking, non-fasting lipid profile, and glycosylated haemoglobin (HBA1C). Bilateral distal 1-cm of the common carotid artery was assessed using ultrasound as per the American Society of Echocardiography Carotid Intima Media Thickness Task Force [18]. All participants had FRS and ASCVD risk scores calculated [25–27]. The proportions of participants categorized as having elevated versus not elevated CVD risk were calculated, with ASCVD predicted risk ≥7.5% and FRS predicted risk ≥10% indicating elevated risk. Agreement in classifying participants as elevated risk for CVD by both cIMT (≥75^th^ percentile and ≥90^th^ percentile, separately, using percentiles based on HIV-negative controls) and the ASCVD score was assessed using McNemar`s Test. Since we hypothesized that ASCVD would classify significantly more participants as elevated CVD risk than FRS, agreement between FRS and cIMT was not assessed. In an exploratory analysis, the optimal cIMT cut off-point that matched ASCVD risk of ≥7.5% was assessed using Youden’s J index; calculated as J = sensitivity + specificity– 1. The maximum value of J may be used to determine an optimal cut-off in scenarios where the costs associated with false positives and false negatives are equal. This index was used along with the receiver operating characteristic (ROC) and corresponding area under this curve (AUC). A historical cohort of 224 HIV-negative controls, mean age 37(±5) years, from the same population with cIMT data was available to define the population’s cIMT 75^th^ percentile cut-off point.

All participants provided written informed consent. The Botswana Ministry of Health Research & Development Committee, Princess Marina Hospital Ethics Committee and Brigham & Women`s Hospital / Massachusetts General Hospital Institutional Review Board all approved the study.

## Results

### Cohort baseline characteristics

Among the 78 participants eligible for ASCVD calculation, 34 (44%) were females with a mean age of 45 years. All participants in this cohort were virally suppressed with mean HIV disease and ART exposure duration of 10.9 and 9.3 years respectively. Baseline demographic, CVD risk factors, and HIV-associated factors are summarized in Table 1 for these 78 eligible participants as well as the 130 participants who were ineligible to have ASCVD calculated.

**Table 1:** Demographics and clinical characteristic of study participants.

*Demographics*	HIV-infected Patients, n = 208	Eligible for ASCVD calculation- 78 (38%)	Ineligible for ASCVD calculation- 130 (62%)
Sex (Female)	114 (55%)	44 (56%)	50 (38%)
Age in years, mean (SD)^a^	39 (5)	44.3 (2.9)	36.2 (3.3)
30–39 years	122 (59%)	N/A	122 (94%)
40–50 years	86 (41%)	78 (100%)	8 (6%)
*Cardiovascular risk factors*			
Cigarette Smoking			
Ever	71 (34%)	32 (41%)	39 (30%)
Current	15(7%)	5 (6%)	10 (8%)
Pack Years^a^	4.7 (6.7)	6.6 (9.2)	3.2 (3)
Diabetes Mellitus- N (%)	1 (0%)	0	1 (1%)
Glycosylated hemoglobin (%)^a^	5.3 (0.5)	5.4 (0.5)	5.3 (0.6)
Hypertension-N (%)	36 (17%)	21(27%)	15 (12%)
Systolic Blood Pressure (mmHg)^a^	130.3 (15.7)	136.5 (15.8)	126.5 (14.4)
Diastolic Blood Pressure (mmHg)^a^	85.1 (12.4)	90.6 (11.7)	81.8 (11.7)
Chronic Kidney Disease- N (%)	7 (3%)	4 (5%)	3 (2%)
Dyslipidaemia- N (%)	17 (8%)	4 (5%)	13 (10%)
Total cholesterol (mmol/L)^a^	4.7 (1.1)	4.8 (1.1)	4.7 (1.2)
LDL-cholesterol (mmol/L)^a^	2.9 (1.0)	2.9 (0.9)	2.9 (1.0)
HDL-cholesterol (mmol/L)^a^	1.4 (0.5)	1.4 (0.5)	1.5 (0.4)
Triglycerides (mmol/L)^a^	1.4(1.1)	1.6 (1.3)	1.2 (0.8)
*Family History*			
Myocardial Infarction- N (%)	2 (1%)	1 (1%)	1 (1%)
Stroke- N (%)	24 (12%)	15 (19%)	9 (7%)
*Medications*			
Anti-hypertensive–N (%)	36 (17%)	21 (27%)	15 (12%)
HMG Co-A inhibitors	10 (5%)	N/A	10 (8%)
Fibrates	1 (0%)	1 (1%)	0
*Anthropometric Data*			
Waist-hip ratio: [N(%)]			
F≥0.85	38 (49%)	12 (71%)	26 (43%)
M≥0.90	21 (33%)	11 (41%)	10 (25%)
*HIV-parameters*			
HIV Disease Duration (years)^a^	10.1 (3.2)	10.9 (3.3)	9.6 (3.1)
Duration on ART^a^	8.6 (2.7)	9.3 (2.1)	8.2 (2.9)
Nadir CD4 count(cells/ul)^a^	126 (99)	90 (67)	147 (109)
Baseline CD4 count (cells/ul)^a^	133 (105)	98 (69)	153 (117)
Current CD4 count (cells/ul)^a^	564 (231)	532 (235)	582 (228)
Proportion with undetectable VL	208 (100%)	78 (100%)	130 (100%)
Time since VL <400 copies/ml (months)	3.1 (1.9)	3.2 (1.8)	3 (2)
Current NRTI exposure			
Zidovudine	93 (45%)	38 (49%)	55 (42%)
Tenofovir	108 (52%)	36 (46%)	72 (55%)
Stavudine	0	0	0
Abacavir	6 (3%)	2 (3%)	4 (3%)
Lamivudine	100 (48%)	42 (54%)	58 (45%)
Patients on NNRTI-based ART	155 (75%)	58 (74%)	97 (75%)
Patients on PI-based ART	52 (25%)	19 (24%)	33 (25%)

Abbreviations: HIV, human immune deficiency virus; SD, standard deviation; ACE, angiotensin converting enzyme; HMG-Co A, 3-hydroxy-3-methylglutaryl-coenzyme A; CVD, cardiovascular disease; LDL, low density lipoprotein; HDL, high density lipoprotein; VL, viral load; NNRTI, non-nucleoside reverse transcriptase inhibitor; PI, protease inhibitor; N/A, not applicable

All values are count (N) and associated percentage, mean (standard deviations), unless denoted otherwise

Baseline CD4 count; last recorded CD4 count prior to ART initiation

Nadir CD4 count; lowest recorded CD4 count in the medical record

### Predicted 10 year CVD risk by ASCVD and FRS

Among the 208 HIV-infected participants with cIMT results, 130 participants did not have an ASCVD risk score calculated for primary prevention of CVD: 122 were less than 40 years old (ASCVD risk score not applicable); of those greater than 40 years old, 6 were already on statin therapy, one had prior diagnosis of diabetes mellitus and one had total cholesterol < 100. The mean predicted 10-year CVD risk for the cohort eligible for risk score calculation (n = 78) was 4% by ASCVD versus 1.97% by FRS. ASCVD classified more participants at elevated CVD risk as compared to FRS (14.1% versus 2.6% respectively, McNemar`s p = 0.01).

### Correlation between ASCVD risk and cIMT

Of the 78 HIV-infected participants with ASCVD scores, cIMT led to categorization of 63 (80.8%) as low CVD risk and 15 (19.2%) as elevated CVD risk (using the cIMT cut-off point determined from a cohort of 65 similar HIV-negative participants between 40 and 50 years old). ASCVD categorized similar proportion of participants as elevated CVD risk compared with cIMT using the ≥75^th^ percentile as a cutoff: 14.1% versus 19.2%, respectively (McNemar’s p = 0.34, Table 2). When the criterion for elevated CVD risk by cIMT was increased to 90^th^ percentile, 76 (97.4%) participants were categorized as low CVD risk versus 2 (2.6%) as high CVD risk by cIMT. Applying the higher cIMT criteria for elevated CVD risk, significantly fewer participants were categorized as elevated risk by ASCVD versus cIMT: 14.1% versus 2.6%, respectively (McNemar`s p = 0.02, Table 3).

**Table 2:** Correlation between ASCVD and cIMT <75^th^ percentile versus ≥75^th^ percentile.

		cIMT		
		Low risk (<75^th^ percentile), N (%)	Elevated risk (≥75^th^ percentile), N (%)	Total, N (%)
ASCVD	Low risk (<7.5%)	60 (76.9)	7 (9.0)	67 (85.9)
	Elevated risk (≥7.5%)	3 (3.8)	8(10.2)*	11 (14.1)
	Total	63 (80.8)	15(19.2)	78 (100%)

Correlation between ASCVD and cIMT <75^th^ percentile versus ≥75^th^ percentile in categorizing HIV-infected participants as low versus elevated risk for CVD

*McNemar exact p-value = 0.34 for agreement between ASCVD & cIMT

**Table 3:** Correlation between ASCVD and cIMT <90^th^ percentile versus ≥90^th^.

		cIMT		
		Low risk (90^th^ percentile), N (%)	Elevated risk (≥90^th^ percentile), N (%)	Total, N (%)
ASCVD	Low risk (<7.5%)	65 (83.3)	2 (2.6)	67 (85.9)
	Elevated risk (≥7.5%)	11 (14.1)	0 (0)*	11 (14.1)
	Total	76 (97.4)	2 (2.6)	78 (100%)

Correlation between ASCVD and cIMT <90^th^ percentile versus ≥90^th^ percentile in categorizing HIV-infected participants as low versus elevated risk for CVD

*McNemar`s exact p-value = 0.02 for agreement between ASCVD & cIMT

We used Youden’s J to calculate the optimal cut point to be the 81^st^ percentile for cIMT, based on ASCVD score ≥7.5% (sensitivity = 72.7% and specificity = 88.1% with AUC 0.82, 95% Mann-Whitney CI: 0.66–0.99). This 81^st^ percentile categorized participants identically to our original 75^th^ percentile cut-off based on HIV-uninfected controls within the 40–50 year age band (cIMT = 0.698mm and 0.707mm for 81^st^ percentile among HIV-infected ASCVD eligible participants versus and 75^th^ percentile among HIV-uninfected controls between 40 and 50 years old, respectively).

## Discussion

In this pilot study of CVD risk prediction in sub-Saharan Africa, using the ACC/AHA CVD risk equation, we demonstrated that among HIV-infected patients age 40–50 years with viral suppression, there was relatively good agreement in risk classification between the ASCVD risk score and cIMT, a validated marker of subclinical atherosclerosis, in classifying participants at high risk for CVD. Agreement in risk classification by the ASCVD risk score and cIMT lost significance with a higher and less clinically relevant cut-point of cIMT of 90^th^ percentile was applied, suggesting that ASCVD algorithm may appropriately to classify patients in the high risk category at least based on observed sub-clinical atherosclerosis.

The relevance of CVD risk prediction tools developed for the general population is unclear in both HIV-infected populations and in sub-Saharan African settings. When the ASCVD risk score has been evaluated in special populations for whom a tailored CVD risk score has not been developed, it has been found to have moderate agreement with surrogate markers of CVD such as cIMT [19]. The Mediators of Atherosclerosis study of South Asians living in America (MASALA) clinical cohort of 849 South Asian adults between 40–75 years old living in San Francisco Bay and greater Chicago showed good agreement between the ASCVD risk score and cIMT. Similarly, in a Korean cohort of 201 adults, high risk classification by ASCVD correlated with high CVD risk classification by cIMT [20]. However, this agreement has not been consistent across studies. In a US population of patients with head and neck cancer, ASCVD under-estimated the proportion of patients classified high risk by the ASCVD when compared with cIMT [28]. While radiation may have induced excess atherosclerosis that could not be predicted by ASCVD, but is clearly detectable on cIMT measurement, this study highlights the utility of cIMT in identifying atherosclerosis induced by novel mechanisms other than traditional CVD risk factors, as is likely the case in our study population.

Our direct comparison of ASCVD to FRS in categorizing patient as elevated CVD risk was important to perform as neither risk prediction rule was developed for HIV-infected patients in SSA. In our cohort, ASCVD classified more patients as elevated CVD risk than FRS as has been observed when these two prediction equations are compared among geographically diverse general populations beyond the US general population [29–32] and some HIV-specific patient population studies [12] but not all [11, 13]. It is possible that the difference in categorizing patients at elevated risk by ASCVD versus FRS in out cohort may reflect the findings that the ASCVD may “over-estimate” risk, even in the general population. We would expect this effect to be more significant among a younger HIV-infected patient population in whom traditional CVD risk factors do not seem to be the main drivers of observed CVD risk such as occurrence of stroke among HIV-infected patients in the SSA setting [22, 23]. Ultimately, either ASCVD or FRS will need to be assessed for the ability to categorize HIV-infected patients who experience CVD end-points such as stroke in SSA as predicted high CVD risk.

Like other imaging modalities, cIMT provides a direct measure of observed subclinical atherosclerosis that result from a combination of risk factors present in the population. In contrast, ASCVD provides an estimate of predicted CVD risk based on traditional CVD risk factors only. Applying the ASCVD risk equation to our cohort of HIV-infected participants, similar proportion of patients were classified as having elevated predicted CVD risk using the risk score versus using an estimate of subclinical atherosclerosis measured by cIMT. Despite this concordance, the finding that nearly ten percent of patients at elevated risk by cIMT measurement were classified as low risk by risk score suggests that a component of CVD risk observed on carotid imaging in our study may be due to increasingly recognized HIV-specific novel risk factors for CVD such as inflammation [33–36] and immune dysregulation [37, 38], among other mechanistic factors, especially in relation to relatively younger HIV-infected patients as was the case in our study [39]. Our data suggests that the ASCVD score may fail to identify HIV-infected patients at higher CVD risk (based on our surrogate CVD endpoint of cIMT). Applying an even higher threshold for classification of high risk by ASCVD of ≥10%, as was recently recommended by the US Preventative Services Task Force [40], is likely to accurately classify even fewer HIV-infected patients at elevated CVD risk.

The main limitations of our study are the small sample size and cross-sectional design. Larger heterogeneous HIV cohorts in SSA followed longitudinally for clinical CVD endpoints such as strokes and myocardial infarctions would provide a better assessment of utility of ASCVD in identifying patients at high CVD risk, and potentially assess the contribution of HIV-specific factors to the total predicted CVD. Using hard CVD clinical end-points such as stroke or myocardial infarction would be more robust measures for assessing CVD risk prediction model calibration and discrimination than subclinical carotid-intima media thickness, as the equations were not developed to evaluate cIMT as an outcome. Thus, we do not provide formal measures of discrimination or calibration. Further, given the strong effect of age on risk of CVD, future work should study HIV-infected patients over 50 years old However, our results provide a rigorous evaluation of CVD risk prediction equations in the sub-Saharan African setting, where hard CVD endpoints are not routinely available, by using carotid atherosclerosis as a surrogate CVD end point and comparing CVD risk classification.

Our results suggest that the ASCVD risk score is reasonably accurate in classifying patients in terms of CVD risk in HIV-infected adults age 40–50 in SSA when using subclinical atherosclerosis as the CVD endpoint. In light of these findings, the ASCVD risk score may be cautiously used among HIV-infected patients in SSA pending further validation of traditional CVD risk prediction tools in SSA using hard clinical endpoints. Developing accurate strategies for CVD risk assessment and primary CVD risk reduction will be an important priority as HIV-infected individuals in sub-Saharan Africa age. Accurate identification of HIV-infected patients who will benefit the most from statin therapy will efficiently guide national programs in SSA in considering resource allocation to reduce the burden of CVD-associated mortality and morbidity versus other urgent needs for HIV-infected populations.
